# Supplementary material for: Bovine milk consumption affects the transcriptome of porcine adipose stem cells: Do exosomes play any role?
Source: PLoS One. 2024 Dec 20;19(12):e0302702. doi: 10.1371/journal.pone.0302702 (PMC11661651; doi:10.1371/journal.pone.0302702)
Supplement: S2 File — Fig 1. Trough used to provide milk to the piglets; Table 1 and Fig 2. Data and results of the transfection of adipose stem cells with various transfection agents; Figs 3 and 4. Images of the adipose stem cells differentiated in adipocytes and osteocytes; Fig 5. Analysis of exosomal markers from exosomes isolated form milk and plasma of pigs; Fig 6. Transcript abundance of mesenchymal and porcine-specific adipose stem cells markers in ASC; Fig 7. Volcano plot of the DEG in between ASC from pigs supplemented with milk vs. ASC of milk supplemented with isocaloric maltodextrin solution. (DOCX) [file pone.0302702.s002.docx]

**Supplementary Material: Bovine Milk Consumption Affects the Transcriptome of Porcine Adipose Stem Cells: do Exosomes Play any Role?**

**
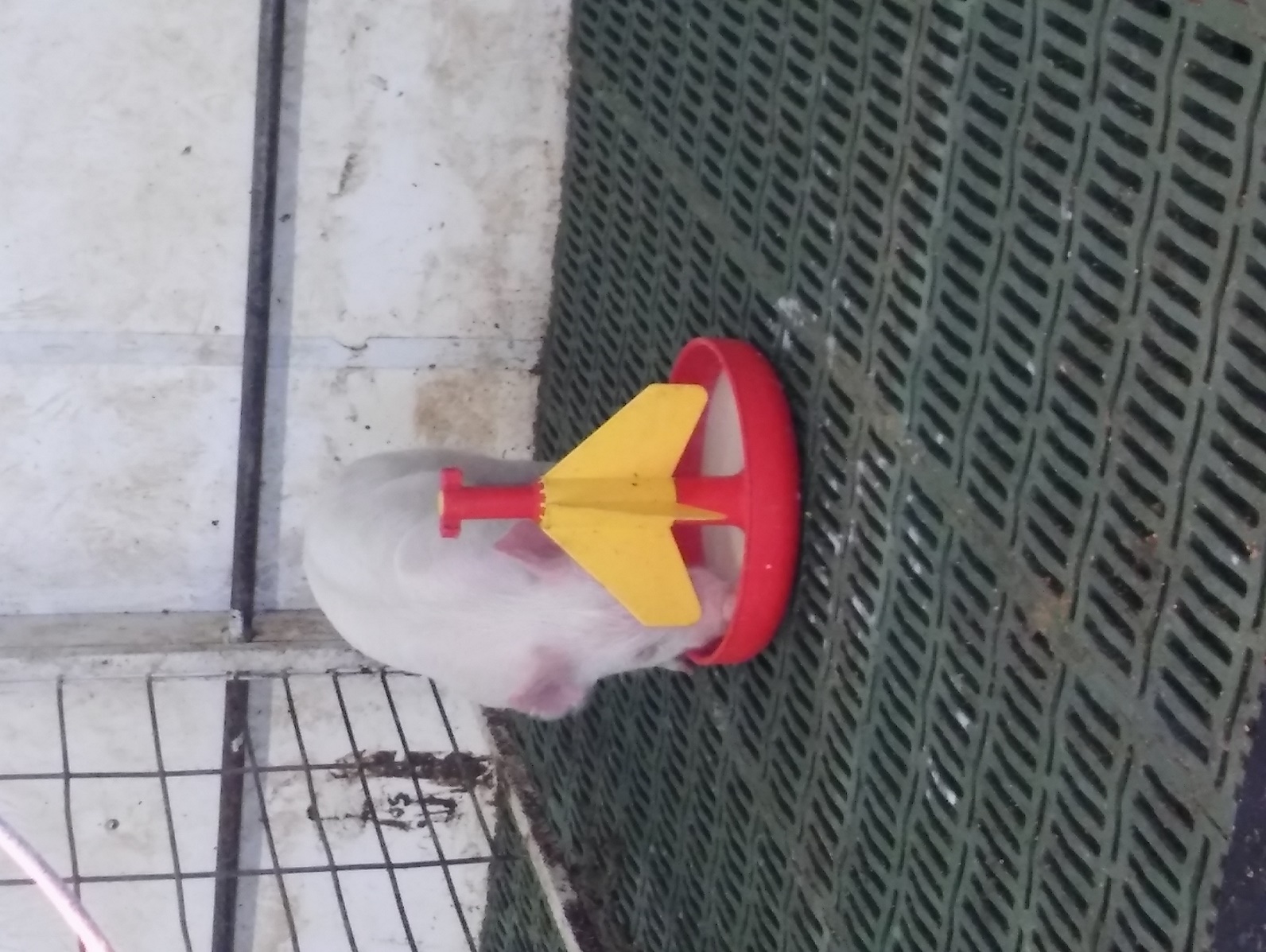
**

**Supplementary Figure 1.** Piglet consuming milk.

**In-vitro transfection of plasmid into ASC**

*Materials and Methods*

**Supplementary Table 1.** **Complete list of the various transfection reagents and various conditions used to determine transfection efficiency for MAC-T and ASC**

| Transfection Reagent | μL Reagent/ Well | μg DNA/Well |
| --- | --- | --- |
| Lipofectamine 3000 | 0.15  0.15  0.30  0.30 | 100  200  100  200 |
| Lipofectamine Stem | 0.30  0.30  0.40  0.40 | 100  200  100  200 |
| EndoFectin | 0.10  0.10  0.20  0.20 | 50  100  50  100 |
| TransFectin | 0.35  0.35  0.60  0.60 | 100  150  100  150 |
| JetPRIME | 0.20  0.30  0.40 | 100  150  100 |
| Transfex | 0.25  0.25  0.50  0.50 | 125  250  125  250 |
| Effectene | 1.25  2.5  5.0 | 50  100  200 |
| PEI-NLS Peptide | 10  10  20  40 | 50  100  50  50 |

To test ASC transfection efficiency, we used Passage 2 ASCs and utilized Bovine Mammary Epithelial Cells (MAC-T) as our positive control^[1]^. 24 hours before transfection, ASCs and MAC-T were plated in a 96-well plate at a density of 10,000 cells/well and 7,500 cells/well, respectively. Cells were kept in DMEM containing 10% FBS, 10000 U/mL of Pennstrep, and 3% amphotericin and incubated at 37oC with 5% CO2. Tested transfection reagents were Lipofectamine Stem (cat # STEM00001, Invitrogen, USA), Lipofectamine 3000 (cat # L3000015, Invitrogen, USA), JetPRIME (cat # 114-01, PolyPlus, USA), TransfeX (cat # ACS-4005, ATCC, USA ), Effectene (cat # 301425, Qiagen, USA), Endofectin (cat # EFM1004-01-S, GeneCopoeia, USA), Transfectin (cat # 170-3350, BIO-RAD, USA), and Polyethylenimine conjugated with the nuclear localization signal peptide (PEI-NLS)^[2]^. Each transfection complex utilized Green Fluorescent Protein (GFP) DNA as a positive indicator of successful transfection. Reagent/DNA conditions are described in **Supplementary Table 1**. Before adding each transfection complex, DMEM was removed and replaced with 50μL of serum-free OptiMEM. Transfection complexes were made according to manufacturer protocol, added to each well, and incubated for ~2 hours. After this, 100μL of OptiMEM containing 10% FBS was added to each well. After incubating for an additional 24 hours, all wells were stained with Hoechst stain (cat# 62249, Thermo Scientific, USA), and images of stained/transfected cells were obtained via fluorescence microscopy (Leica DMI6000B). CellProfiler was used to count stained cells, and transfection efficiency was defined as the % of the number of cells expressing GFP divided by the number of total viable cells.

*Results*

Results are shown in **Suppl. Figure 2**. The highest average transfection efficiency in MAC-T was observed using TransfeX reagent (23.45%) while the lowest was observed with Effectene (0.25%). Likewise, the highest average transfection efficiency in ASC was observed using Lipofectamine Stem (2.25%) and lowest was observed with Effectene (0.07%). Transfection efficiency was significantly (p < 0.05) greater in MAC-T compared to ASC when TransfeX, Lipofectamine 3000, Lipofectamine Stem, JetPRIME, and PEI-NLS were used. No significant differences were found between cell types for Effectene, Endofectin, and Transfectin.

**
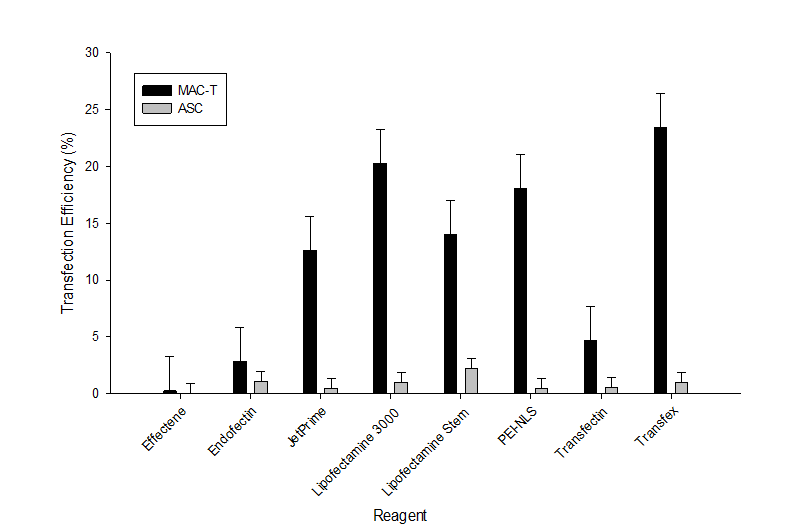
Supplementary Figure 2.** Transfection efficiency of the various transfection reagents in ASC (adipose-derived stem cells) and MACT (bovine alveolar mammary epithelial cells).^]^

*Discussion*

Gene transfer and stem cell therapy offers exciting potential for treating genetic disorders. The pluripotent nature of mesenchymal stem cells provides great flexibility for various targets of genetic therapy^[3,4]^. To evaluate the potential of adipose stem cells’ ability to transfer genetic material, we tested popular transfection reagents at many concentrations with various levels of GFP DNA. We observed maximum transfection efficiency of 2.25%, this is similar to data collected by Razmkhal and coworkers, who achieved a maximum transfection efficiency of 1.3% using liposomal transfection methods^[4]^. To elucidate further transfection methods, we tested a PEI-NLS peptide following protocol described by Park and colleagues^[2]^. We obtained an average transfection efficiency of ~0.5%, significantly lower than the ~23% achieved by Park and coworkers while using the same conditions^[2]^. ASC proved to be very difficult to transfect and using our current methods we were unable to achieve suitable transfection efficiencies for further analyses^[1]^.

**
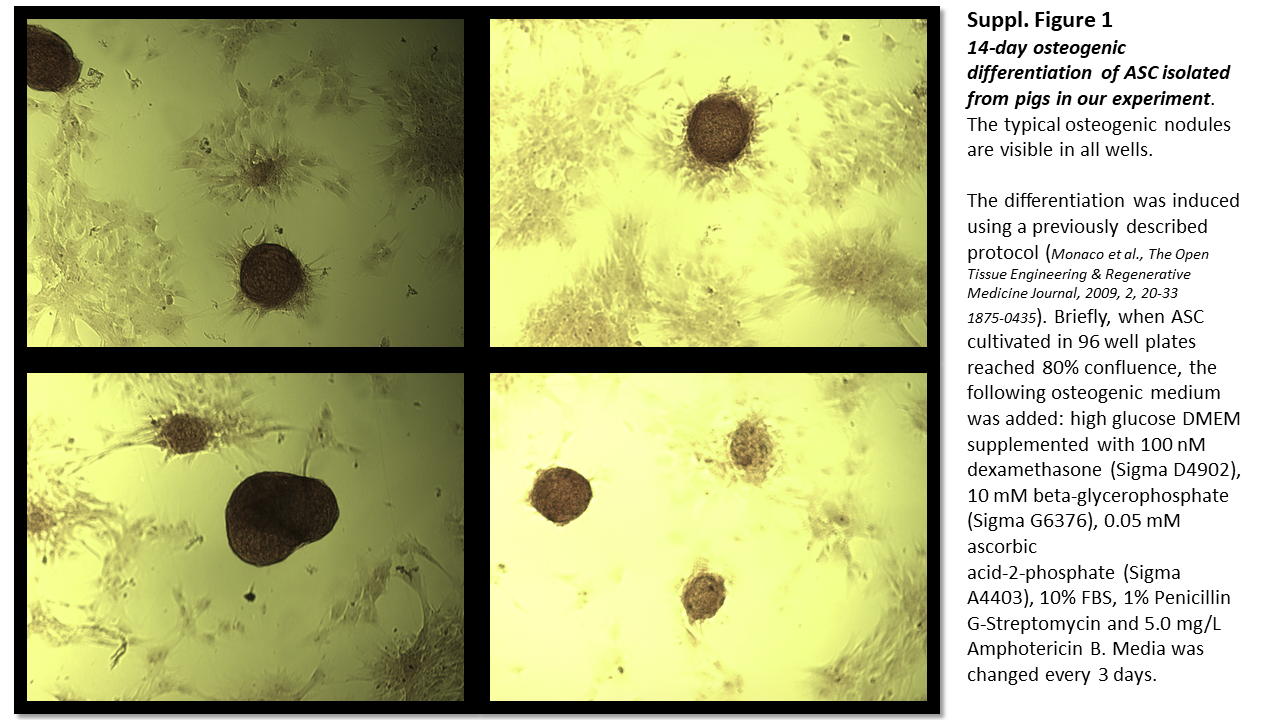
**

**Supplementary Figure 3.** 14-day osteogenic differentiation of ASC isolated from pigs. The typical osteogenic nodules are visible in all wells. The differentiation was induced using a previously described protocol (Monaco et al., The Open Tissue Engineering & Regenerative Medicine Journal, 2009, 2, 20-331875-0435). Briefly, when ASC cultivated in 96 well plates reached 80% confluence, the following osteogenic medium was added: high glucose DMEM supplemented with 100 nM dexamethasone (Sigma D4902), 10 mM beta-glycerophosphate (Sigma G6376), 0.05 mM ascorbic acid-2-phosphate (Sigma A4403), 10% FBS, 1% Penicillin G-Streptomycin and 5.0 mg/L Amphotericin B. Media was changed every 3 days.

**
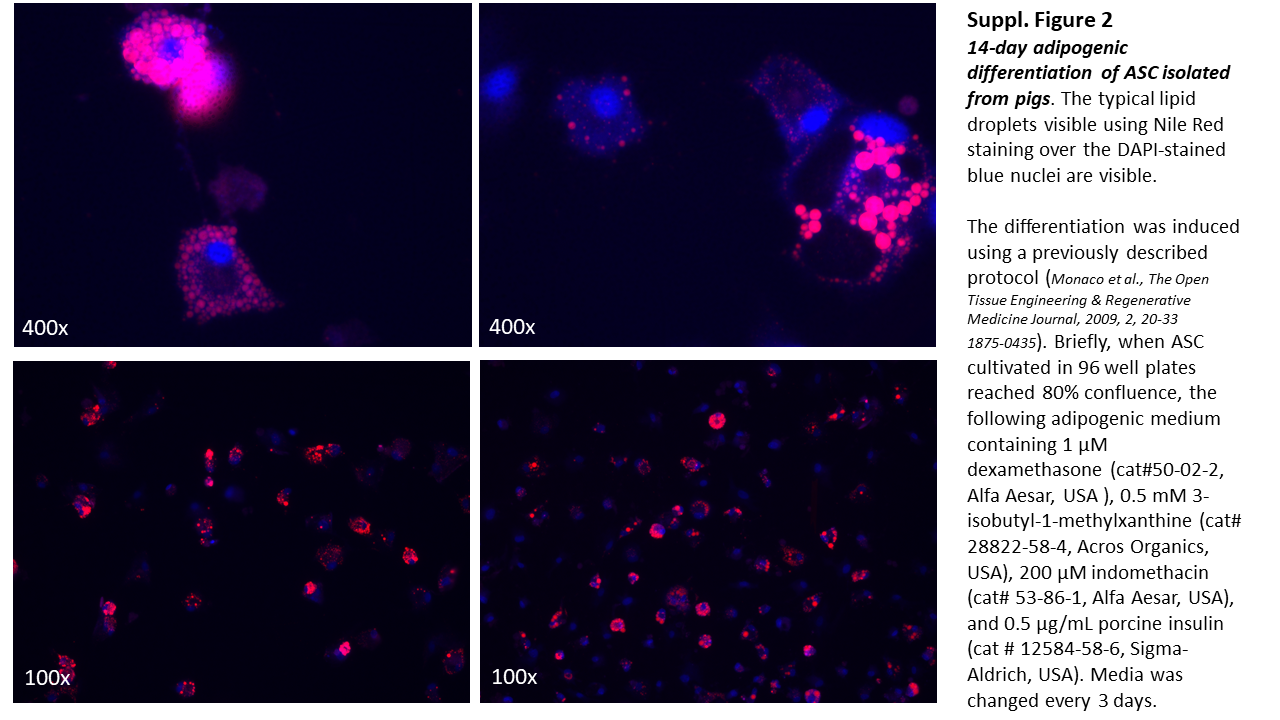
**

**Supplementary Figure 4.** 14-day adipogenic differentiation of ASC isolated from pigs. The typical lipid droplets are visible using Nile Red staining over the DAPI-stained blue nuclei. The differentiation was induced using a previously described protocol (Monaco et al., The Open Tissue Engineering & Regenerative Medicine Journal, 2009, 2, 20-331875-0435). Briefly, when ASC cultivated in 96 well plates reached 80% confluence, the following adipogenic medium containing 1 µM dexamethasone (cat#50-02-2, Alfa Aesar, USA ), 0.5 mM 3-isobutyl-1-methylxanthine (cat# 28822-58-4, Acros Organics, USA), 200 µM indomethacin (cat# 53-86-1, Alfa Aesar, USA), and 0.5 µg/mL porcine insulin (cat # 12584-58-6, Sigma-Aldrich, USA). Media was changed every 3 days.


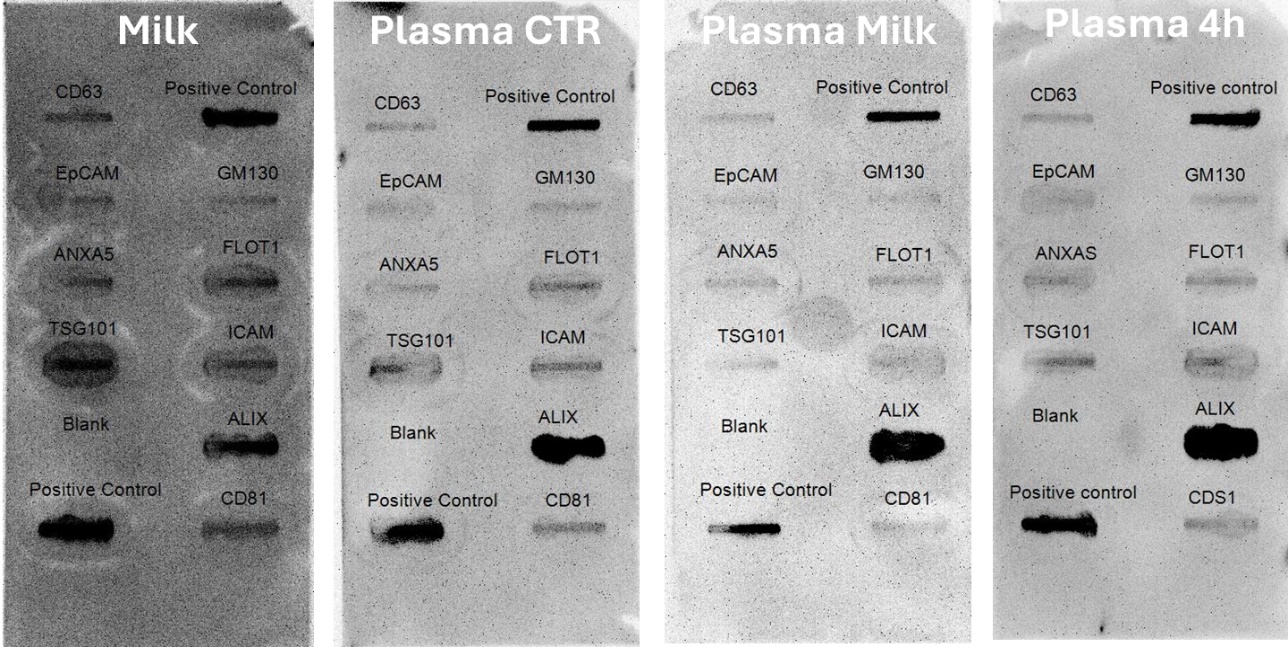


**Supplementary Figure 5.** Eight protein markers for exosomes (CD63 = Tetraspanin; EpCAM = Epithelial cell adhesion molecule; often found in cancer-derived exosomes; ANXA5 = Annexin A5; TSG101 = Tumor susceptibility gene 101; FLOT1 = Flotillin-1; ICAM1 Intercellular adhesion molecule 1; ALIX = Programmed cell death 6 interacting protein; CD81 = Tetraspanin), plus two positive controls, one background spot, and the GM130 = Cis-golgi matrix protein – control for cellular contamination in exosome preparation were measured via Exo-Check antibody array kit in pool of exosomes isolated from: cow milk (Milk), plasma of the 6 piglets fed the control diet (Plasma CTR), plasma of the 6 piglets fed milk (Plasma Milk), and plasma of three piglets collected 4 hours after consumption of milk (Plasma 4h).

**Supplementary Figure 6.** Transcription of mesenchymal positive and negative markers, both as general markers ^[5]^ (left graph) or pig-specific adipose stem cell markers^[6]^ (right graph). Transcription of three internal controls usually used in Western blot is shown for comparison.

**
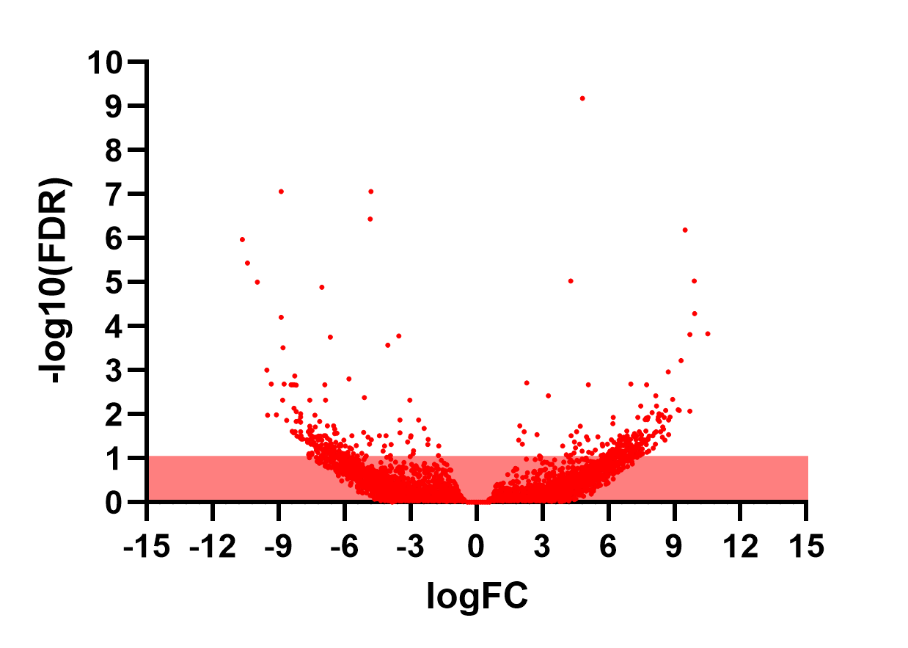
**

**Supplementary Figure 7.** Volcano plot of the whole transcriptome of adipose stem cells isolated from pig-fed milk or maltodextrin (control).

**REFERENCES**

1. Osorio JS, Bionaz M. Plasmid transfection in bovine cells: Optimization using a realtime monitoring of green fluorescent protein and effect on gene reporter assay. *Gene* 2017;626:200-8.

2. Park E, Cho HB, Takimoto K. Effective gene delivery into adipose-derived stem cells: transfection of cells in suspension with the use of a nuclear localization signal peptide-conjugated polyethylenimine. *Cytotherapy* 2015;17:536-42.

3. Abdul Halim NS, Fakiruddin KS, Ali SA, Yahaya BH. A comparative study of non-viral gene delivery techniques to human adipose-derived mesenchymal stem cell. *Int J Mol Sci* 2014;15:15044-60.

4. Razmkhah M, Jaberipour M, Ghaderi A. Downregulation of MMP2 and Bcl-2 in Adipose Derived Stem Cells (ASCs) following Transfection with IP-10 Gene. *Avicenna J Med Biotechnol* 2014;6:27-37.

5. Mildmay-White A, Khan W. Cell Surface Markers on Adipose-Derived Stem Cells: A Systematic Review. *Curr Stem Cell Res Ther* 2017;12:484-92.

6. Garcia GA, Oliveira RG, Dariolli R, Rudge MVC, Barbosa AMP, et al. Isolation and characterization of farm pig adipose tissue-derived mesenchymal stromal/stem cells. *Braz J Med Biol Res* 2022;55:e12343.
