# Supplementary material for: Bovine milk consumption affects the transcriptome of porcine adipose stem cells: Do exosomes play any role?
Source: PLoS One. 2024 Dec 20;19(12):e0302702. doi: 10.1371/journal.pone.0302702 (PMC11661651; doi:10.1371/journal.pone.0302702)
Supplement: Attachment — Submitted filename: Review 23.08.24.docx [file pone.0302702.s010.docx]

Review manuscript no. PONE-D-24-14376

Bovine Milk Consumption Affects the Transcriptome of Porcine Adipose Stem Cells: Do

Exosomes Play any Role?

Katherine Swanson, Jimmy Bell, David Hendrix, Duo Jiang, Brandon Batty, Melanie

Hanlon and Massimo Bionaz

**General comment**

The investigators studied the nutrigenomic effects of cow´s milk consumption on

the transcriptome of adipocyte stem cells (ASCs) of porcine subcutaneous adipose tissue after the piglet´s lactation period, with special attention to bovine milk exosomal microRNAs (miRs).

In detail: ASCs isolated from 19-20 week old piglets supplemented for 11 weeks with 750 mL of raw whole cow´s milk tended (P=0.10) to have a higher amount of ASCs isolated per gram of subcutaneous adipose tissue and a higher amount of CFU and ASC proliferation than control piglets fed an isocaloric protein-free maltodextrin solution.

Over 500 genes were differentially expressed (DEG) in ASCs isolated from bovine milk-fed vs. maltodextrin-fed control piglets. Bioinformatic analysis of DEG indicated an inhibition of the immune, neuronal, and endocrine systems and insulin-related pathways in ASCs of milk-fed piglets. Surprisingly, bioinformatic data point to an inhibition of the mTOR pathway in ASCs isolated from piglets consuming milk despite increased ASC proliferation. Experimental direct determination such as pS6K1 expression – as a measure of mTORC1 activity – have not been presented.

The investigators did not detect any exosomal miR or mRNA transfer from bovine milk to porcine-circulating plasma exosomes when investigating three 5-week-old Yorkshire Hampshire piglets implying that milk´s nutrigenomic effect on ASCs is not attributed to miRs in milk exosomes as observed after the lactation period.

The key message of the study is that cow´s milk consumption increased ASC numbers and ASC proliferation. However, the biological contribution of milk exosomal miRs on the epigenetic regulation of ASCs during lactation before and after weaning remains controversial and requires further investigations.

**Specific comments**

**Major flaws regarding the study design**

**The selected time period**

To assess the impact of cow´s milk on adipocyte stem cell (ASC) proliferation in the porcine system, the physiology of porcine lactation and weaning has to be matched more closely. The nursing time of domestic pigs is 3-4 weeks, whereas wild pigs provide milk up to 16 weeks.

The study started with piglets 8- to 9 weeks of age exposed to cow´s milk for further 11 weeks. Thus, the study may have missed a critical early window of postnatal milk-exosome-controlled ASC proliferation.

**Selected animals and postnatal time periods**

The reviewer wonders why the investigators used Duroc-Berkshire male piglets 8-9 weeks of age (n=6) for their milk challenge experiment but 5-week-old Yorkshire Hampshire piglets (n=3) for their exosome miR studies. Thus, the genetic background of the animals, the number of studied animals and timing of the sampling differ substantially, an avoidable mismatch for the later translation of obtained results.

Nevertheless, the use of 5 week old piglets may have compromised intestinal milk exosome and miR uptake and miR transmission into the circulatory system during this late stage of lactation (differences of colostrum milk exosomes versus mature milk exosomes). Differences in intestinal permeability and exosome composition and quantity have to be considered.

In 3–6 days old human neonates, intestinal permeability decreases in both term and preterm neonates.

van Elburg RM, Fetter WP, Bunkers CM, Heymans HS. Intestinal permeability in relation to birth weight and gestational and postnatal age. Arch Dis Child Fetal Neonatal Ed. 2003 Jan;88(1):F52-5. doi: 10.1136/fn.88.1.f52. PMID: 12496227; PMCID: PMC1755997.

Frazer LC, Good M. Intestinal epithelium in early life. Mucosal Immunol. 2022 Jun;15(6):1181-1187. doi: 10.1038/s41385-022-00579-8. Epub 2022 Nov 15. PMID: 36380094; PMCID: PMC10329854.

According to literature data, the intestinal closure in the piglet, i.e. the dramatic decrease in the transfer of macromolecules from the intestinal epithelium in the blood, is at about 24 h of age.

Ekström GM, Weström BR. Cathepsin B and D activities in intestinal mucosa during postnatal development in pigs. Relation to intestinal uptake and transmission of macromolecules. Biol Neonate. 1991;59(5):314-21. doi: 10.1159/000243365. PMID: 1714775.

Clarke RM, Hardy RN. Histological changes in the small intestine of the young pig and their relation to macromolecular uptake. J Anat. 1971 Jan;108(Pt 1):63-77. PMID: 5543214; PMCID: PMC1234227.

Ekström GM, Weström BR, Telemo E, Karlsson BW. The uptake of fluorescein-conjugated dextran 70,000 by the small intestinal epithelium of the young rat and pig in relation to macromolecular transmission into the blood. J Dev Physiol. 1988 Jun;10(3):227-33. PMID: 2464019.

Colostrum exosome transfer during the first hours and days of postnatal life may have already stabilized the intestinal permeability barrier limiting later transfer of mature milk exosomes.

Aarts J, Boleij A, Pieters BCH, Feitsma AL, van Neerven RJJ, Ten Klooster JP, M'Rabet L, Arntz OJ, Koenders MI, van de Loo FAJ. Flood Control: How Milk-Derived Extracellular Vesicles Can Help to Improve the Intestinal Barrier Function and Break the Gut-Joint Axis in Rheumatoid Arthritis. Front Immunol. 2021 Jul 28;12:703277. doi: 10.3389/fimmu.2021.703277. PMID: 34394100; PMCID: PMC8356634.

Tong L, Zhang S, Liu Q, Huang C, Hao H, Tan MS, Yu X, Lou CKL, Huang R, Zhang Z, Liu T, Gong P, Ng CH, Muthiah M, Pastorin G, Wacker MG, Chen X, Storm G, Lee CN, Zhang L, Yi H, Wang JW. Milk-derived extracellular vesicles protect intestinal barrier integrity in the gut-liver axis. Sci Adv. 2023 Apr 14;9(15):eade5041. doi: 10.1126/sciadv.ade5041. Epub 2023 Apr 12. PMID: 37043568; PMCID: PMC10096581.

Guo MM, Zhang K, Zhang JH. Human Breast Milk-Derived Exosomal miR-148a-3p Protects Against Necrotizing Enterocolitis by Regulating p53 and Sirtuin 1. Inflammation. 2022 Jun;45(3):1254-1268. doi: 10.1007/s10753-021-01618-5. Epub 2022 Jan 29. PMID: 35091894.

Melnik BC, Stremmel W, Weiskirchen R, John SM, Schmitz G. Exosome-Derived MicroRNAs of Human Milk and Their Effects on Infant Health and Development. Biomolecules. 2021 Jun 7;11(6):851. doi: 10.3390/biom11060851. PMID: 34200323; PMCID: PMC8228670.

Samuel M, Chisanga D, Liem M, Keerthikumar S, Anand S, Ang CS, Adda CG, Versteegen E, Jois M, Mathivanan S. Bovine milk-derived exosomes from colostrum are enriched with proteins implicated in immune response and growth. Sci Rep. 2017 Jul 19;7(1):5933. doi: 10.1038/s41598-017-06288-8. PMID: 28725021; PMCID: PMC5517456.

**Weaning stress**

It has recently been emphasized that weaning stress often causes changes in the morphology and function of the small intestine of piglets, disrupts digestion and absorption capacity, destroys intestinal barrier function, and ultimately leads to reduced feed intake, increased diarrhea rate, and growth retardation, a point that may be considered as well as the exosome study falls into this period.

Tang X, Xiong K, Fang R, Li M. Weaning stress and intestinal health of piglets: A review. Front Immunol. 2022 Nov 24;13:1042778. doi: 10.3389/fimmu.2022.1042778. PMID: 36505434; PMCID: PMC9730250.

At present, systematic studies investigating the time course of colostrum/milk exosome uptake from birth (time of colostrum exposure) to later periods of lactation (time of mature milk exposure) are missing. Individuals with inflammatory bowel diseases and leaky gut exhibit increased intestinal permeability, a constellation modifying milk exosome kinetics and uptake.

Michielan A, D'Incà R. Intestinal Permeability in Inflammatory Bowel Disease: Pathogenesis, Clinical Evaluation, and Therapy of Leaky Gut. Mediators Inflamm. 2015;2015:628157. doi: 10.1155/2015/628157. Epub 2015 Oct 25. PMID: 26582965; PMCID: PMC4637104.

**Significant differences in protein intake during the intervention period**

The total protein content of the first porcine colostrum (16.65%) is approximately three times the level in porcine mature milk at the end of lactation (5.83%).

Csapó J, Martin TG, Csapó-Kiss ZS, Házas Z. Protein, fats, vitamin and mineral concentrations in porcine colostrum and milk from parturition to 60 days. Intern Dairy J 1966, 6 (8–9): 881-902; <https://doi.org/10.1016/0958-6946(95)00072-0>

After weaning pigs are physiologically not exposed to milk proteins and milk protein-derived signaling as well as milk exosomes and their potential miR-regulated gene expression.

During the intervention period of this study, piglets received 750 mL whole cow´s milk/day with 3,6% protein concentration resulting in 27 g milk protein uptake/day.

In comparison to the protein-free maltodextrin control, the milk-group received an additional total milk protein amounts of 2 kg providing substantial intake of essential branched-chain amino acids (BCAA) during the intervention period.

Only balancing the caloric intake between the milk group and the maltodextrin group is an oversimplified approach overlooking critical metabolic and epigenetic effects of BCAA-mTORC1 signaling.

BCAAs, enriched in milk proteins, are key activators of insulin and IGF-1 secretion as well as BCAA-mediated mTORC1 activation.

The authors provide no concrete data on mTORC1 activation such as the expression of the mTORC1 downstream target phosphorylated S6K1 (pS6K1). A Western blot analysis of pS6K1 should be provided to control the real impact of milk intake on mTORC1-S6K1 activation in ASCs.

High mTORC1-S6K1 signaling has been linked to increased adipogenesis by high protein formula feeding of human infants.

Melnik BC. Excessive Leucine-mTORC1-Signalling of Cow Milk-Based Infant Formula: The Missing Link to Understand Early Childhood Obesity. J Obes. 2012;2012:197653. doi: 10.1155/2012/197653. Epub 2012 Mar 19. PMID: 22523661; PMCID: PMC3317169.

Melnik BC. The potential mechanistic link between allergy and obesity development and infant formula feeding. Allergy Asthma Clin Immunol. 2014 Jul 22;10(1):37. doi: 10.1186/1710-1492-10-37. PMID: 25071855; PMCID: PMC4112849.

Exposure of infants to high protein formula is an accepted explanation of pediatrics for increased postnatal adipogenic programming by formula feeding.

Luque V, Closa-Monasterolo R, Escribano J, Ferré N. Early Programming by Protein Intake: The Effect of Protein on Adiposity Development and the Growth and Functionality of Vital Organs. Nutr Metab Insights. 2016 Mar 20;8(Suppl 1):49-56. doi: 10.4137/NMI.S29525. PMID: 27013888; PMCID: PMC4803318.

Haschke F, Grathwohl D, Detzel P, Steenhout P, Wagemans N, Erdmann P. Postnatal High Protein Intake Can Contribute to Accelerated Weight Gain of Infants and Increased Obesity Risk. Nestle Nutr Inst Workshop Ser. 2016;85:101-9. doi: 10.1159/000439492. Epub 2016 Apr 18. PMID: 27088337.

**mTORC1 activation is required for differentiation of mesenchymal stem cells to ASCs**

Of note, activated mTORC1 plays a central role in the differentiation of mesenchymal stem cells to ASCs.

Xiang X, Zhao J, Xu G, Li Y, Zhang W. mTOR and the differentiation of mesenchymal stem cells. Acta Biochim Biophys Sin (Shanghai). 2011 Jul;43(7):501-10. doi: 10.1093/abbs/gmr041. Epub 2011 Jun 2. PMID: 21642276.

Zhou J, Li D, Wang F. Assessing the function of mTOR in human embryonic stem cells. Methods Mol Biol. 2012;821:361-72. doi: 10.1007/978-1-61779-430-8_23. PMID: 22125078.

Furthermore, the mTOR signaling pathway is also involved in the regulation of high glucose-induced intramuscular adipogenesis in porcine muscle satellite cells

Yue T, Yin J, Li F, Li D, Du M. High glucose induces differentiation and adipogenesis in porcine muscle satellite cells via mTOR. BMB Rep. 2010 Feb;43(2):140-5. doi: 10.5483/bmbrep.2010.43.2.140. PMID: 20193134.

On the other hand, mTORC1 inhibition via rapamycin suppresses human adipocyte differentiation.

Bell A, Grunder L, Sorisky A. Rapamycin inhibits human adipocyte differentiation in primary culture. Obes Res. 2000 May;8(3):249-54. doi: 10.1038/oby.2000.29. PMID: 10832768.

Studies in mice revealed that S6K1 plays a critical role in early adipocyte differentiation and is involved in the commitment of embryonic stem cells to early adipocyte progenitors.

Carnevalli LS, Masuda K, Frigerio F, Le Bacquer O, Um SH, Gandin V, Topisirovic I, Sonenberg N, Thomas G, Kozma SC. S6K1 plays a critical role in early adipocyte differentiation. Dev Cell. 2010 May 18;18(5):763-74. doi: 10.1016/j.devcel.2010.02.018. PMID: 20493810; PMCID: PMC2918254.

**Cow milk feeding of young mice enhances pS6K1 expression in white adipose tissue**

The reviewer wonders why the authors overlooked a very similar experimental approach presented in young mice allowing to consume long-term commercial cow´s milk.

Bar Yamin H, Barnea M, Genzer Y, Chapnik N, Froy O. Long-term commercial cow's milk consumption and its effects on metabolic parameters associated with obesity in young mice. Mol Nutr Food Res. 2014 May;58(5):1061-8. doi: 10.1002/mnfr.201300650. Epub 2013 Dec 23. PMID: 24550222.

Compared to the control group (chow with water supplementation), the levels of pS6K1 protein in white murine adipose tissue were significantly higher in the groups of mice having access to low-fat- and whole milk as quantified by Western blotting.

Recent evidence indicates that pS6K1 enters the nucleus leading to H2BS36 phosphorylation and recruitment of EZH2 to H3, which mediates H3K27 trimethylation. This blocks WNT gene expression, inducing the upregulation of PPARγ and CEBP/alpha promoting adipogenesis.

Yi SA, Um SH, Lee J, Yoo JH, Bang SY, Park EK, Lee MG, Nam KH, Jeon YJ, Park JW, You JS, Lee SJ, Bae GU, Rhie JW, Kozma SC, Thomas G, Han JW. S6K1 Phosphorylation of H2B Mediates EZH2 Trimethylation of H3: A Determinant of Early Adipogenesis. Mol Cell. 2016 May 5;62(3):443-452. doi: 10.1016/j.molcel.2016.03.011. Epub 2016 Apr 14. PMID: 27151441; PMCID: PMC5325705.

Yi SA, Han J, Han JW. Epigenetic role of nuclear S6K1 in early adipogenesis. BMB Rep. 2016 Aug;49(8):401-2. doi: 10.5483/bmbrep.2016.49.8.116. PMID: 27439608; PMCID: PMC5070725.

Juan AH, Sartorelli V. S6K1ing to ResTOR Adipogenesis with Polycomb. Mol Cell. 2016 May 5;62(3):325-326. doi: 10.1016/j.molcel.2016.04.018. Erratum in: Mol Cell. 2016 Nov 17;64(4):850. doi: 10.1016/j.molcel.2016.10.031. PMID: 27153531.

Wang H. Role of EZH2 in adipogenesis and obesity: Current state of the art and implications - A review. Medicine (Baltimore). 2022 Sep 9;101(36):e30344. doi: 10.1097/MD.0000000000030344. PMID: 36086687; PMCID: PMC10980444.

Suppression of WNT expression by excessive milk protein-mTORC1-S6K1 signaling may thus explain ASC proliferation as observed in the presented study. Accordingly, the manuscript could be substantially improved by presenting mRNA and protein expression of pS6K1, WNT6, WNT10A and WNT10B.

WNT signaling maintains preadipocytes in an undifferentiated state through inhibition of the adipogenic transcription factors CCAAT/enhancer binding protein alpha (C/EBPalpha) and peroxisome proliferator-activated receptor gamma (PPARgamma). When WNT signaling in preadipocytes is prevented these cells differentiate into adipocytes.

Ross SE, Hemati N, Longo KA, Bennett CN, Lucas PC, Erickson RL, MacDougald OA. Inhibition of adipogenesis by Wnt signaling. Science. 2000 Aug 11;289(5481):950-3. doi: 10.1126/science.289.5481.950. PMID: 10937998.

Yuan Z, Li Q, Luo S, Liu Z, Luo D, Zhang B, Zhang D, Rao P, Xiao J. PPARγ and Wnt Signaling in Adipogenic and Osteogenic Differentiation of Mesenchymal Stem Cells. Curr Stem Cell Res Ther. 2016;11(3):216-25. doi: 10.2174/1574888x10666150519093429. PMID: 25986621.

D'Alimonte I, Lannutti A, Pipino C, Di Tomo P, Pierdomenico L, Cianci E, Antonucci I, Marchisio M, Romano M, Stuppia L, Caciagli F, Pandolfi A, Ciccarelli R. Wnt signaling behaves as a "master regulator" in the osteogenic and adipogenic commitment of human amniotic fluid mesenchymal stem cells. Stem Cell Rev Rep. 2013 Oct;9(5):642-54. doi: 10.1007/s12015-013-9436-5. PMID: 23605563; PMCID: PMC3785124.

de Winter TJJ, Nusse R. Running Against the Wnt: How Wnt/β-Catenin Suppresses Adipogenesis. Front Cell Dev Biol. 2021 Feb 9;9:627429. doi: 10.3389/fcell.2021.627429. PMID: 33634128; PMCID: PMC7900430.

**FTO – a epigenetic key player in adipogenesis**

FTO plays a key role in adipocyte development and adipogenesis.

Merkestein M, Sellayah D. Role of FTO in Adipocyte Development and Function: Recent Insights. Int J Endocrinol. 2015;2015:521381. doi: 10.1155/2015/521381. Epub 2015 Dec 16. PMID: 26788058; PMCID: PMC4695642.

FTO mediates cell-autonomous effects on adipogenesis and adipocyte lipid content by regulating gene expression via 6mA DNA modifications.

Martin Carli JF, LeDuc CA, Zhang Y, Stratigopoulos G, Leibel RL. FTO mediates cell-autonomous effects on adipogenesis and adipocyte lipid content by regulating gene expression via 6mA DNA modifications. J Lipid Res. 2018 Aug;59(8):1446-1460. doi: 10.1194/jlr.M085555. Epub 2018 Jun 22. PMID: 29934339; PMCID: PMC6071765.

FTO activates mTORC1.

Gulati P, Cheung MK, Antrobus R, Church CD, Harding HP, Tung YC, Rimmington D, Ma M, Ron D, Lehner PJ, Ashcroft FM, Cox RD, Coll AP, O'Rahilly S, Yeo GS. Role for the obesity-related FTO gene in the cellular sensing of amino acids. Proc Natl Acad Sci U S A. 2013 Feb 12;110(7):2557-62. doi: 10.1073/pnas.1222796110. Epub 2013 Jan 28. PMID: 23359686; PMCID: PMC3574930.

It has been shown that FTO promotes adipogenesis through inhibition of the WNT/β-catenin signaling in porcine intramuscular preadipocytes.

Chen X, Luo Y, Jia G, Liu G, Zhao H, Huang Z. FTO Promotes Adipogenesis through Inhibition of the Wnt/β-catenin Signaling Pathway in Porcine Intramuscular Preadipocytes. Anim Biotechnol. 2017 Oct 2;28(4):268-274. doi: 10.1080/10495398.2016.1273835. Epub 2017 Mar 7. PMID: 28267420

Thus, high protein access during the postnatal period may promote early adipogenesis via FTO-mTORC1-S6K1-activation suppressing WNT signaling. The manuscript would thus improve substantially by providing data on FTO expression in ASCs before and after milk challenges.

Melnik BC, Weiskirchen R, Stremmel W, John SM, Schmitz G. Risk of Fat Mass- and Obesity-Associated Gene-Dependent Obesogenic Programming by Formula Feeding Compared to Breastfeeding. Nutrients. 2024 Jul 28;16(15):2451. doi: 10.3390/nu16152451. PMID: 39125332; PMCID: PMC11314333.

Apparently, the milk protein is the critical activator of mTORC1-S6K1-WNT-controlled ASC proliferation providing the key epigenetic mechanism promoting the early steps of adipogenesis. As shown by the authors, milk miR signaling appears to play a less significant epigenetic role after the weaning period.

**Methods**

**The characterization of ASC populations is missing**

The International Society for Cellular Therapy (ISCT) proposed a minimum set of criteria for identifying cells as mesenchymal stem cells: (1) adherence to plastic; (2) expression of CD73, CD90, and CD105 but lack of expression of CD45, CD34, CD14 or CD11b, CD79a or CD19, and HLA-DR surface molecules; and (3) potential to differentiate into pre-adipocytes, chondrocytes, and osteoblasts.

Dominici M, Le Blanc K, Mueller I, Slaper-Cortenbach I, Marini F, Krause D, Deans R, Keating A, Prockop Dj, Horwitz E. Minimal criteria for defining multipotent mesenchymal stromal cells. The International Society for Cellular Therapy position statement. Cytotherapy. 2006;8(4):315-7. doi: 10.1080/14653240600855905. PMID: 16923606.

The authors should present the surface marker characterization of their selected populations of ASCs. ASCs can be identified in adipose tissue by the expression of markers such as Integrin family members, CD44, and ICAM-1/CD54. The following markers should be positive: CD90, CD44, CD29, CD105, CD13, CD34, CD73, CD166, CD10, CD49e and CD59. The following markers should be negative: CD31, CD45, CD14, CD11b, CD19, CD56 and CD146.

Mildmay-White A, Khan W. Cell Surface Markers on Adipose-Derived Stem Cells: A Systematic Review. Curr Stem Cell Res Ther. 2017;12(6):484-492. doi: 10.2174/1574888X11666160429122133. PMID: 27133085.

Ong WK, Tan CS, Chan KL, Goesantoso GG, Chan XH, Chan E, Yin J, Yeo CR, Khoo CM, So JB, Shabbir A, Toh SA, Han W, Sugii S. Identification of specific cell-surface markers of adipose-derived stem cells from subcutaneous and visceral fat depots. Stem Cell Reports. 2014 Feb 6;2(2):171-9. doi: 10.1016/j.stemcr.2014.01.002. PMID: 24527391; PMCID: PMC3923222.

There is not “one” adipocyte stem cell, but a heterogeneity of ASCs differing in function and progenitor cell differentiation. Especially, there is a difference in gene expression between visceral fat- (V-ASC) and subcutaneous fat-derived ASCs (S-ASC). S-ASCs had higher capacity to proliferate and to differentiate into adipogenic lineage than V-ASCs.

Kim B, Lee B, Kim MK, Gong SP, Park NH, Chung HH, Kim HS, No JH, Park WY, Park AK, Lim JM, Song YS. Gene expression profiles of human subcutaneous and visceral adipose-derived stem cells. Cell Biochem Funct. 2016 Dec;34(8):563-571. doi: 10.1002/cbf.3228. Epub 2016 Nov 17. PMID: 27859461.

Cleal L, Aldea T, Chau YY. Fifty shades of white: Understanding heterogeneity in white adipose stem cells. Adipocyte. 2017 Jul 3;6(3):205-216. doi: 10.1080/21623945.2017.1372871. Epub 2017 Sep 12. PMID: 28949833; PMCID: PMC5638386.

Hwang I, Kim JB. Two Faces of White Adipose Tissue with Heterogeneous Adipogenic Progenitors. Diabetes Metab J. 2019 Dec;43(6):752-762. doi: 10.4093/dmj.2019.0174. PMID: 31902145; PMCID: PMC6943255.

Liao X, Zhou H, Deng T. The composition, function, and regulation of adipose stem and progenitor cells. J Genet Genomics. 2022 Apr;49(4):308-315. doi: 10.1016/j.jgg.2022.02.014. Epub 2022 Feb 28. PMID: 35240306.

Ferrero R, Rainer P, Deplancke B. Toward a Consensus View of Mammalian Adipocyte Stem and Progenitor Cell Heterogeneity. Trends Cell Biol. 2020 Dec;30(12):937-950. doi: 10.1016/j.tcb.2020.09.007. Epub 2020 Oct 23. PMID: 33148396.

Nahmgoong H, Jeon YG, Park ES, Choi YH, Han SM, Park J, Ji Y, Sohn JH, Han JS, Kim YY, Hwang I, Lee YK, Huh JY, Choe SS, Oh TJ, Choi SH, Kim JK, Kim JB. Distinct properties of adipose stem cell subpopulations determine fat depot-specific characteristics. Cell Metab. 2022 Mar 1;34(3):458-472.e6. doi: 10.1016/j.cmet.2021.11.014. Epub 2022 Jan 11. PMID: 35021043.

As the study did not characterize the transcriptome of V-ASCs in milk-fed piglets, information of cow milk´s impact on V-ASCs is missing. This is however of key importance because visceral fat promotes pro-inflammatory signaling, a critical aspect for obesity-related pathologies. This fact should at least be discussed in the manuscript.

**The influence of ASC-derived exosomes is not considered**

ASCs exhibit an active secretome and deliver exosomes and exosomal miRs that may as well be involved in the process of adipogenesis.

Wang J, Wu H, Peng Y, Zhao Y, Qin Y, Zhang Y, Xiao Z. Hypoxia adipose stem cell-derived exosomes promote high-quality healing of diabetic wound involves activation of PI3K/Akt pathways. J Nanobiotechnology. 2021 Jul 7;19(1):202. doi: 10.1186/s12951-021-00942-0. PMID: 34233694; PMCID: PMC8261989.

Zhang Y, Zouboulis CC, Xiao Z. Exosomes from adipose-derived stem cells activate sebocytes through the PI3K/AKT/SREBP-1 pathway to accelerate wound healing. Cell Tissue Res. 2024 Jun;396(3):329-342. doi: 10.1007/s00441-024-03872-z. Epub 2024 Feb 27. PMID: 38411945; PMCID: PMC11144157.

Zhang W, Bai X, Zhao B, Li Y, Zhang Y, Li Z, Wang X, Luo L, Han F, Zhang J, Han S, Cai W, Su L, Tao K, Shi J, Hu D. Cell-free therapy based on adipose tissue stem cell-derived exosomes promotes wound healing via the PI3K/Akt signaling pathway. Exp Cell Res. 2018 Sep 15;370(2):333-342. doi: 10.1016/j.yexcr.2018.06.035. Epub 2018 Jun 28. PMID: 29964051.

An Y, Lin S, Tan X, Zhu S, Nie F, Zhen Y, Gu L, Zhang C, Wang B, Wei W, Li D, Wu J. Exosomes from adipose-derived stem cells and application to skin wound healing. Cell Prolif. 2021 Mar;54(3):e12993. doi: 10.1111/cpr.12993. Epub 2021 Jan 17. PMID: 33458899; PMCID: PMC7941238.

**Discussion**

As the authors observed an increase in ASC proliferation in subcutaneous fat tissue, which could not explained by epigenetic effects of mature milk exosomes, they should present an alternative explanation for the observed effect. It is the reviewer´s suggestion that milk protein signaling enhancing mTORC1-S6K1 signaling is involved in the suppression of WNT signaling promoting the observed ASC proliferation. The mice study of Bar Yamin et al. has to be discussed in this context.

Arderiu et al. have identified that miR-29b-3p in both S-ASCs and V-ASCs and miR-424-5p and miR-378a-3p in S-ASCs are involved in differentiation into endothelial cell (EC)-like cells. These miRs modulate their pro-angiogenic effects on ASCs by targeting *FGFR1*, *NRP2*, *MAPK1*, and *TGF-β2*, and the MAPK signaling pathway. miR-29b-3p upregulation contributes to ASCs' differentiation into EC-like cells by directly targeting *TGFB2* in both S-ASCs and V-ASCs. Moreover, their results reveal that, independent of S-ASCs' origin (obese/lean), the upregulation of miR-378a-3p and the downregulation of miR-424-5p inhibit *MAPK1* and overexpress *FGFR1*and *NRP2*, respectively.

Arderiu G, Civit-Urgell A, Díez-Caballero A, Moscatiello F, Ballesta C, Badimon L. Differentiation of Adipose Tissue Mesenchymal Stem Cells into Endothelial Cells Depends on Fat Depot Conditions: Regulation by miRNA. Cells. 2024 Mar 14;13(6):513. doi: 10.3390/cells13060513. PMID: 38534357; PMCID: PMC10969675.

This reference should be discussed in the context of miR signaling.

Concerning cow´s milk consumption and insulin resistance, the study of Hoppe et al. should be discussed showing increased insulin secretion and insulin resistance in prepubertal boys after cow milk intake.

Hoppe C, Mølgaard C, Vaag A, Barkholt V, Michaelsen KF. High intakes of milk, but not meat, increase s-insulin and insulin resistance in 8-year-old boys. Eur J Clin Nutr. 2005 Mar;59(3):393-8. Doi: 10.1038/sj.ejcn.1602086. PMID: 15578035.

Overfeeding of mice by decreasing litter size during the early postnatal period increased insulin, and IGF-1 levels, accelerated growth, increased body weight, impaired glucose tolerance and increased arterial blood pressure in comparison to normally fed mice of normal litter size.

Kappeler L, De Magalhaes Filho C, Leneuve P, Xu J, Brunel N, Chatziantoniou C, Le Bouc Y, Holzenberger M. Early postnatal nutrition determines somatotropic function in mice. Endocrinology. 2009 Jan;150(1):314-23. doi: 10.1210/en.2008-0981. Epub 2008 Sep 18. PMID: 18801897.

**Minor errors:**

Line 558: Check reference 10.1002/jnr.23138, which does not fit. The paper deals with neural stem cells and not ASCs.

Line 640: “5-year old piglets” / 5-week old piglets

**Final comment:**

The manuscript requires a major revision presenting direct determinations of FTO, pS6K1 and WNT gene expression at the RNA and protein levels. We cannot only rely on bioinformatic data analysis. As outlined above published literature evidence contradicts the presented observations and conclusions based on bioinformatic data analyses.

Furthermore, ASCs used in the experiments should be characterized providing surface markers expression.

The discussion of manuscript needs a further refinement considering available literature data on ASC epigenetics and biology.
